# Intriguing role of the Golgi apparatus in astrocyte function: Implications for disorders

**DOI:** 10.4103/NRR.NRR-D-25-00342

**Published:** 2025-08-13

**Authors:** Martina Polenghi, Elena Restelli, Elena Taverna, Laura Tapella

**Affiliations:** 1 Human Technopole, Neurogenesis Research Center, Milan, Italy; 2 Department of Pharmaceutical Sciences, Università del Piemonte Orientale “Amedeo Avogadro”, Novara, Italy

**Keywords:** astrocytes, congenital glycosylation disorders, glia, Golgi apparatus, homeostatic function, local protein translation, neurodegeneration, neuroinflammation, neuronal development, secretory pathway

## Abstract

Cell function has a tight relationship with cell architecture. Distribution of proteins to the correct compartment is one of the functions of the traffic pathway through the Golgi apparatus. The others are to ensure proper protein folding, the addition of post-translational modifications, and delivering to intracellular and extracellular destinations. Astrocytes are fundamental homeostatic cells, controlling multiple aspects of the central nervous system physiology, such as ion balance, nutrients, blood flow, neurotransmitters, and responses to insults. Astrocytes are polarized cells, and, such as neurons, extensively use the secretory pathway for secreting factors and exposing functional receptors, channels, and transporters on the plasma membrane. In this review, we will underline the importance of studying the Golgi apparatus and the secretory pathway in astrocytes, based on the possible tight connection between the Golgi apparatus and astrocytes’ homeostatic function. Given the topic of this review, we will provide examples mostly about the Golgi apparatus structure, function, localization, and its involvement in astrocytes’ homeostatic response, with an insight into congenital glycosylation disorders, as an example of a potential future field in the study of astrocyte homeostatic failure and Golgi apparatus alteration.

## Conventional Protein Trafficking by the Golgi Apparatus

Cell function and shape have a tight relationship, guaranteed by the traffic pathway, compartmentalization, and distribution of organelles and molecular components such as proteins and lipids. Polarized cells show an even closer relation between their function and intracellular compartmentalization. The secretory pathway is extensively used in neurons and astrocytes to secrete factors and to deliver functional receptors, channels, and transporters on the plasma membrane, allowing cell–cell communication and interaction. Indeed, traffic organelles, most notably the endoplasmic reticulum (ER) and Golgi apparatus (GA), play a role in determining cellular features. Neurons and astrocytes show an even closer bond between their function, intracellular trafficking, and compartmentalization of organelles, ensuring cell–cell interaction.

### Golgi apparatus structure and secretory pathway

**Figure 1:**
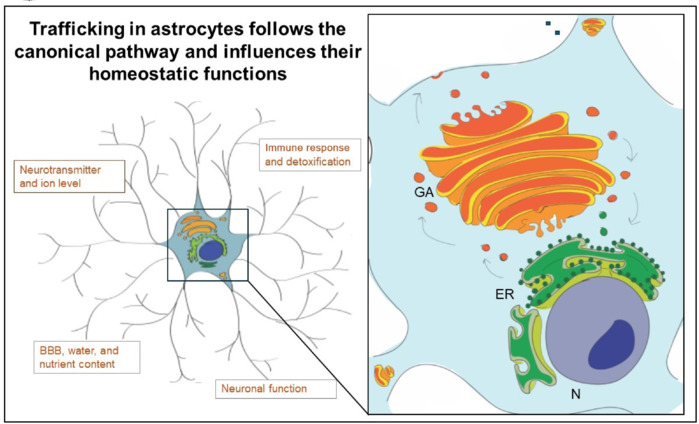
Intracellular trafficking in astrocytes and homeostatic function. The GA is depicted in orange, while the ER is represented in green. Trafficking in astrocytes follows the canonical pathway, where a protein is first translated and post-translationally modified in the ER and subsequently delivered to the GA, where further processing occurs. While exiting from the GA, the protein is packed into vesicles and sorted to its final destination, either inside the cell, on the plasma membrane, or in the extracellular environment. BBB: Blood–brain barrier; ER: endoplasmic reticulum; GA: Golgi apparatus; N: nucleus.

GA is one of the main hubs of the conventional secretory pathway. GA is formed by cisternal membranes, packed together to form the Golgi stack. These stacks are divided into different regions: the cis-, medial Golgi, and trans-Golgi, where vesicles bud to deliver cargo to their destination. In vertebrates, the adjacent stacks are interconnected by tubular membranes, conferring the classical ribbon-like shape. Vesicles bud and fuse along the cisternae to ensure the correct delivery of structural proteins and glycosylation enzymes in the GA (Glick and Luini, 2011; D’Souza et al., 2020; Park et al., 2021b; **Figure 1**).

The conventional secretory pathway starts when nascent proteins are delivered to the ER while, or right after, being synthesized. From there, they are packed into COPII vesicles, they exit at specific ER exit sites, and they first fuse with the ER-Golgi Intermediate Compartment before being delivered to the GA. Here, proteins reach further maturation, processing, and sorting while passing from the cis to medial to trans-Golgi. Finally, from the trans-Golgi network, depending on their intended function and final localization, they exit packed into vesicles that will (1) go to the plasma membrane and either be inserted there or secreted in the extracellular environment; (2) be delivered to endosomes, lysosomes or back to the Golgi or the ER in COPI vesicles (Park et al., 2021b).

Golgi matrix proteins, such as Golgins and Golgi reassembly stacking proteins, together with SNAREs, kinases, methyltransferases, ATPases, and GTPases, support and maintain Golgi structure and mediate the intense trafficking of vesicles (Volchuk et al., 2004; Park et al., 2021b).

### Mutations associated with Golgi apparatus proteins

Mutations in Golgi proteins are characteristics of CNS disorders affecting trafficking, ion balance, pH, and N- and O-glycosylation.

For example, GM130 is a cis-Golgi matrix protein that maintains the structure and function of the Golgi apparatus. It plays a role in vesicle tethering and transport and even impacts centrosome morphology and mitotic spindle formation (Nakamura, 2010). GM130 mutations in mouse neurons lead to Golgi disruption, impaired trafficking pathways, and the development of progressive cerebellar atrophy (Liu et al., 2021).

SNARE proteins play a significant role in the Golgi apparatus, mediating the specificity of membrane fusion and cargo transport within the organelle. They facilitate both anterograde (ER to Golgi) and retrograde (Golgi to ER) trafficking of vesicles (Malsam and Söllner, 2011; Jahn et al., 2024). SNARE mutations have instead been linked to abnormal sorting of neurotransmitter receptors at neuronal synapses (Liu et al., 2021).

Importantly, ATPases, resident Golgi proteins involved in maintaining Golgi luminal Ca^2+^, Mn^2+^, and Cu^2+^ ion concentration, affect the optimal activity of glycosyltransferases and cargo trafficking. Mutations in ATPase copper transporting α (ATP7A) result in its mislocalization and impairment of copper response, with the development of Menkes disease, whereas mutations in ATPase copper transporting β (ATP7B) lead to Wilson’s disease development (De Bie et al., 2007; Dringen et al., 2013).

Finally, control of the pH is crucial for GA functionality. Ubiquitin-protein ligase E3A is linked to the elevated pH of GA cisternae with the consequent reduction of protein sialylation observed in the Angelman syndrome mouse model (Kellokumpu, 2019).

In general, it has been observed that mutations to Golgi proteins are linked to N- and O-glycosylation and trafficking defects, involved in the control and maintenance of Golgi pH (Kellokumpu, 2019), and underlining the central role of the GA and Golgi proteins in cells.

## Astrocytes: Homeostatic Function, and Its Defects, and the Requirement of a Tight Control of Protein Localization and Function

Astrocytes are part of the neuroglia, along with oligodendrocytes, oligodendrocyte precursors, and ependymal cells with a neuroepithelial origin. Other neuroglial cells are peripheral glia from neuronal crest and microglia from myeloid derivation. “Astrocyte” describes a large and heterogeneous group of cells with different morphologies and functions. Astrocytes include: (1) radial glia, neuronal stem cells during brain development; (2) protoplastic astrocytes of the grey matter; (3) fibrous astrocytes of the white matter; (4) specialized astrocytes, including velate astrocytes of the cerebellum, marginal astrocytes, pituicytes of the neurohypophysis, Gomori astrocytes in the arcuate nucleus of the hypothalamus and, in the hippocampus, surface-associated astrocytes and perivascular astrocytes; (5) radial astrocytes, which are represented by cerebellar Bergmann glial cells, radial astrocytes in the neurogenic niches, tanycytes in hypothalamus and the spinal cord and Müller retinal glial cells; (6) ependymocytes, choroid plexus cells and retinal pigment epithelial cells. Additionally, other types have been described only in humans and primates (Verkhratsky and Rose, 2020).

### Homeostatic function and its defects

Astrocytes populate the white and grey matter of the CNS. They are fundamental homeostatic cells by controlling multiple aspects of CNS physiology (Parpura and Verkhratsky, 2012). Astrocytes monitor ions, protons, and metal contents, remove neurotransmitters, and supply neurotransmitter precursors. They provide energy, waste metabolic products, and protect from insults and pathogens (Adamsky and Goshen, 2018). Astrocytes contact neuronal soma and synapse with the perisynaptic astrocytic processes (PAPs) (Aboufares El Alaoui et al., 2020; Ko et al., 2023). Moreover, they contribute to glutamatergic and GABAergic neurotransmission by glutamate (GABA)-glutamine shuttle.

Astrocytes are non-excitable cells, but they integrate stimuli, changing cytoplasmic ions, such as Ca^2+^, Na^+^, Cl^−^, and K^+^, and second messengers, such as cyclic adenosine monophosphate. Intracellular spatio-temporal fluctuations of Ca^2+^ and Na^+^ are fundamental for maintaining homeostatic function (Parpura and Verkhratsky, 2012). Indeed, they regulate synaptogenesis, synaptic maturation, isolation, and maintenance through the expression of several astrocyte-specific enzymes and Na^+^-dependent transporter (Lim et al., 2021; Rose and Verkhratsky, 2024).

Ca^2+^ signals are crucial in astrocytes. Initiated by adenosine triphosphate (ATP) and glutamate, they regulate metabolism, proteostasis, and trafficking/ secretion. Ca^2+^ signals have even been associated with protein synthesis and mitochondrial integrity, whose alteration can impact homeostatic functions. Astrocytes exhibit various forms of calcium signaling, described as waves, microdomains, and intrinsic fluctuations. Shaping the complex interplay between astrocytes, neurons, and their environment (Lia et al., 2021; Lim et al., 2021, 2023).

Astrocytes protect the CNS by forming a selective and protective barrier from insults and pathogens at the blood–brain barrier (BBB) (Li et al., 2024). In the CNS, metal content can modulate enzymes and receptors, exerting a positive or negative effect based on concentration. Astrocytes take up, store, or remove metals, such as iron, copper, manganese, and zinc (Li et al., 2024). Astrocytes can adapt and change reversibly, and this property is called astrocyte plasticity. Indeed, astrocytes are fundamental to neuronal maintenance and function, actively overcoming physiological changes (Allen, 2013; Lalo et al., 2021).

By the study of different neurological disorders, it has emerged that astrogliopathology includes a range of astrocyte states, spanning from reactivity and pathological astrocytes to astrocytic degeneration, asthenia, and loss of function (Escartin et al., 2021). Acute or chronic insults, genetically or environmentally acquired, with cell-autonomous or non-cell-autonomous origins, and mechanisms describe the plethora of pathological causes of pathophysiological changes of astrocytes (Verkhratsky et al., 2023). As a result of various lesions of the nervous tissue, astrocytes respond with a characteristic process called reactive astrogliosis (Escartin et al., 2021). This response is part of and basis of neuroinflammation, due to trauma, infection, or autoimmune response. Instead, in neurodegenerative diseases and epilepsy, astrocytes contribute to the pathology with other types of response (Sofroniew and Vinters, 2010; Escartin et al., 2021; Vezzani et al., 2022). In Alzheimer’s and Parkinson’s diseases, the remodeling of astrocytes depends on transcription, protein expression, and secretion, resulting in a loss of homeostatic support. Instead, an asthenic phenotype has been reported in neuropsychiatric diseases. In addition, in aging, astrocyte changes can be observed as atrophy and loss of homeostatic function (Lim et al., 2013, 2016, 2021, 2023; Dematteis et al., 2020; Verkhratsky et al., 2020; Tapella et al., 2021, 2022). To summarize, the astroglial remodeling leads to the gain of new function(s) or loss or upregulation of homeostatic ones, contributing to different neurological disorders and representing a new target for therapy (Verkhratsky et al., 2016).

### Requirement of a tight control of protein localization and function

Homeostatic function of astrocytes needs proper expression, and trafficking of receptors, transporters, and channels, including (a) ATP or gradient-dependent transporters; (b) neurotransmitter and precursor transporters; (c) metabolic-related transporters; (d) metabotropic and ionotropic receptors, Ca^2+^ signaling toolkit; (e) metals transporters; (f) localization of protein at perisynaptic astrocytic processes, and astrocytic endfeet.

#### Adenosine triphosphate or gradient-dependent transporters

Indeed, astrocytes express multiple transporters (Lovatt et al., 2007; Cahoy et al., 2008), such as pumps or ATP-dependent transport or solute carrier transporters dependent on an electrochemical gradient of different ions, with Na^+^ being retained as important (Verkhratsky and Rose, 2020; Rose and Verkhratsky, 2024). Na^+^ involved transports include ionostatic astroglial transporters, such as Na^+^-K^+^ ATPase, Na^+^/HCO3^–^ co-transport, plasma membrane Na^+^/Ca^2+^ exchangers, mitochondrial Na^+^/Ca^2+^ exchanger, Na^+^/H^+^ exchanger, Na^+^/K^+^/Cl^–^ cotransporter.

#### Neurotransmitter and precursor transporters

Astrocytes express neurotransmitter transporters that are Na^+^ dependent, such as excitatory amino acid transporters EAAT1/2, GAT-GABA transporters, glycine transporters GlyT1, adenosine transporters, and monoamine transporters. Astrocytes provide neurotransmitter precursors, or positive modulators, glutamine, and D-serine, respectively. Glutamine transporters, such as sodium-coupled neutral amino acid transporters SNAT3 and SNAT5, and L/D-serine transporters, ASCT2, have been observed in astrocytes (Schell et al., 1995, 1997; Verkhratsky and Rose, 2020).

#### Metabolic-related transporters

Astrocytes sustain neuronal metabolism. They store 50% of the glucose entering the brain and are the only cells that synthesize glucagon. Glucose transporters GLUT1 and SGLT1 are expressed. Monocarboxylate transporters 1 and 4, MCT1 and MCT4, two lactate transporters, have been reported. Lactate is the preferred neuronal energy substrate, and astrocytes can produce and transport it to neurons thanks to that protein complex (Rafiki et al., 2003; Véga et al., 2006; Halestrap, 2012; Kreft et al., 2012; Allen and Messier, 2013).

#### Metabotropic and ionotropic receptors, Ca^2+^ signaling toolkit

Sodium homeostasis and signaling are crucial, but calcium is considered equally relevant. Indeed, astrocytes integrate and respond to neuronal stimulation by generating calcium signals. Calcium signals are triggered by glutamate and ATP stimulation. Activation of receptors results in calcium influx and/or release from intracellular stores. Astrocytes express channels, transporters, pumps, and buffer proteins representing the “Ca^2+^ signaling toolkit” that determine the spatio-temporal nature of calcium signals (Bazargani and Attwell, 2016; Lia et al., 2021, 2023; Lim et al., 2021). The main astrocyte calcium signals are ligand-operated signaling through ionotropic or metabotropic receptors, linked to the release of Ca^2+^ ER and the Golgi apparatus. Metabotropic receptors linked to trimeric G-proteins containing Gαq/11 and all three isoforms of IP3R have been found in astrocytes. Ca^2+^ entry may occur through several ligand-gated channels: glutamate-sensitive N-methyl-D-aspartate receptors, purinergic P2X7 receptors, dopamine D1/D2 receptors, and α7-containing nicotinic acetylcholine receptors. Store-operated Ca^2+^ channels, including Orai and TRP channels and STIM, an ER luminal Ca^2+^ sensor, have been observed (Khakh and Sofroniew, 2015; Lim et al., 2021, 2023; Sherwood et al., 2021). Ca^2+^ and Na^+^ dynamics in astrocytes are tightly coupled, and the coordinating role of Na^+^/Ca^2+^ exchangers in Ca^2+^-Na^+^ signaling interplay has been extensively discussed (Parpura and Verkhratsky, 2012).

#### Metal transporters

Astrocytes can accumulate and release iron. In astrocytes, iron transport is allowed by divalent metal transporter 1, the complex of holo-transferrin and transferrin receptors, ion channels, and alternatively through transient receptor potential canonical channels. Ion channels and the ferroportin-ceruloplasmin cascade can mediate iron release. Moreover, astrocytes provide the antioxidant system to overcome the eventual toxicity of iron (Pelizzoni et al., 2013; Urrutia et al., 2013; Xia et al., 2021; Li et al., 2024).

Many enzymes need copper, which exists in two oxidation states: cuprous (Cu^+^) and cupric (Cu^2+^). Cu^+^ increases at the synapse during depolarization and can modulate ionotropic receptors, such as glutamatergic receptors and purinoceptors. Astrocytes store and accumulate Cu^+^ by Cu transporters Ctr1, divalent metal transporter 1, and several Zrt/IRT-like (ZIP) metal transporters. In the cytosol, glutathione or metallothioneins bind and store Cu^+^. In astrocytes, ATP7A, located in lysosomes and the Golgi complex, regulates the Cu^+^ levels, contributing to various cell processes including peptide amidation, iron oxidation, and, with ATP7B, drives Cu^+^ to mitochondria to be a cofactor for mitochondrial respiration (Tiffany-Castiglioni et al., 2011; Dringen et al., 2013; Pal et al., 2021; Li et al., 2024).

Manganese can be in two oxidation states, Mn^2+^ and Mn^3+^. Astrocytes transport Mn^2+^ by divalent metal transporter 1, zinc transporter, while the transferrin receptor pathway mediates the Mn^3+^ translocation. It has been reported that Mn^2+^ binds to glutamine synthetase, and high levels of Mn^2+^ inhibit glutamate clearance and functional activity of GABA glutamine shuttle (Aschner et al., 1992, 1992; Sidoryk-Wegrzynowicz and Aschner, 2013; Lee et al., 2017; Li et al., 2024).

Moreover, astrocytes can accumulate Zn^2+^. They also express various zinc transporters, such as ZIP14, ZnT3, ZnT1, ZnT3, ZnT4, and ZnT6, that can impact neuron activity and CNS physiology (Alirezaei et al., 2002; Wang et al., 2005; Segawa et al., 2015; Amagai et al., 2023; Li et al., 2024).

#### Localization of protein at perisynaptic astrocytic processes, and astrocytic endfeet

PAPs are specialized, membrane extensions of astrocytes. They cover synapses and are crucial for astrocyte-neuron communication, supporting synaptic function, such as glutamate uptake, and glucose transport (Ko et al., 2023). Indeed, glutamate and glucose transporters, EAAT and GLUT1, have been reported in astrocytes (Chaudhry et al., 1995; Morgello et al., 1995; Sakers et al., 2017; Ko et al., 2023). Moreover, ER and mitochondria organelles are also contained, expressing proteins and allowing PAPs to perform many functions (Aboufares El Alaoui et al., 2020). Local protein translation has been reported, and astrocytes synthesized transporters and receptors at the PAPs, likely being demonstrated for neurons at the synapses (Sakers et al., 2017).

Transporters and channels are densely packed in the astrocytic endfeet, mediating substance exchange through the glia–vascular interface (Deng et al., 2018; Verkhratsky and Pivoriūnas, 2023). In particular, glucose transporter, potassium channel Kir4.1, and water channel aquaporin-4 (AQP4) have been found at BBB, facilitating glucose uptake, potassium buffering, and water transport (Nicchia et al., 2004; Nwaobi et al., 2016). The BBB is a selectively permeable membrane that separates the brain’s microenvironment from the blood circulation, preventing harmful substances and pathogens from entering the brain. The parenchymal part of the BBB is composed of the parenchymal basement membrane and astrocytes. Astrocytes secrete various factors that affect the expression and function of tight junctions, regulating the BBB permeability (Khakh and Sofroniew, 2015; Kriaučiūnaitė et al., 2021; Mills et al., 2022; Verkhratsky and Pivoriūnas, 2023). In summary, astrocyte endfeet maintain BBB integrity. They participate in regulating blood flow and influencing neuronal activity. As well, PAPs are fundamental for astrocyte-synapse interaction.

## Golgi Apparatus in the Physiology and Pathology of Astrocytes

Astrocytes are polarized cells and use the secretory pathway to secrete factors and traffic functional receptors, channels, and transporters, with proper structure and post-translational modification. GA was discovered when studying one of the most well-known secretory cells, neurons. Since then, the polarization of this organelle has been broadly explored (Nakagawa, 2024). Despite the extensive literature on neuronal intracellular trafficking and GA, neurons are not the only cell type of the nervous system involved in secretion. Astrocytes use several alternative secretory pathways to regulate neurogenesis, synaptogenesis, and neuroinflammation (Verkhratsky et al., 2016).

Polarity allows astrocytes to integrate and regulate neuronal networks and BBB signals. Astrocyte changes have been described in several neurodegenerative and neuropsychiatric disorders (Verkhratsky et al. 2023). In astrocytes, Golgi outposts (GOs) are satellite Golgi organelles found in the endfeet. While these outposts are observed in astrocytes, their specific function is still not fully understood (Valenzuela et al., 2020; Kemal et al., 2022).

As secretory cells, astrocytes can release substances at the BBB through their endfeet or directly regulate neuronal function through the secretion of neurotransmitters, neuromodulators, hormones, peptides, metabolic substrates, and growth factors (Verkhratsky et al. 2016).

Furthermore, astrocytes are involved in detoxification and inflammation, thus, the uptake, release, and detection of extracellular compounds are crucial. The broad and complex functions of astrocytes strictly depend on their secretory and metabolic network (Véga et al., 2006; Kreft et al., 2012; Verkhratsky et al., 2016). The metabolic network of astrocytes is composed of 3765 genes, 862 enzymes, 5007 metabolites, and 5659 reactions. About 131 genes, 182 responses, and 275 metabolites are associated with GA, indicating the relevance of this organelle for astrocyte activity (Martín-Jiménez et al., 2017; Deng et al., 2018). Supporting the importance of GA and trafficking in astrocytes, it has been observed that GA fragmentation is present in numerous neurodegenerative diseases such as Parkinson’s disease, Alzheimer’s disease, amyotrophic lateral sclerosis, and stroke (Verkhratsky et al., 2023). Homeostatic function happens through the release and uptake of molecules, transporters, channels, or receptors through the secretory pathway. Thus, we may speculate a link/association of homeostatic functions between the GA and astrocytes.

### Golgi apparatus involvement in homeostatic functions of astrocytes

Astrocytes exert homeostatic activity in the CNS and play a crucial role in neuronal functions. Astrocytes guarantee metabolic support, ions, and neurotransmitter modulation, supporting the extracellular environment and modulating synaptic activity and BBB permeability (Boulay et al., 2017; Adamsky and Goshen, 2018; **Figure 1**). The literature highlighted their role in CNS development, physiology, and pathology in the past two decades. In CNS pathologies, astrocytes undergo complex and variable changes in terms of structure, molecular expression, and function, with unclear meaning to disease outcomes and subcellular molecular processes (Boulay et al., 2017; Garcia-Esparcia et al., 2017; Sakers et al., 2017; Sims et al., 2022; Verkhratsky et al., 2023). Here, we reported some examples of GA involvement relevant to the role of astrocytes in CNS: (a) Golgi apparatus in astrocyte endfeet sustain local translation; (b) Golgi apparatus mediated glycosylation regulates astrocytic functions; (c) Golgi apparatus and glutamate transporter; (d) Golgi apparatus and voltage-gated potassium channels (Kv) localization; (e) Golgi apparatus and metal detoxification; (f) Golgi apparatus and ethanol detoxification; (g) Golgi apparatus is involved in neuroinflammation.

#### Golgi apparatus in astrocyte endfeet sustains local translation

**Figure 2:**
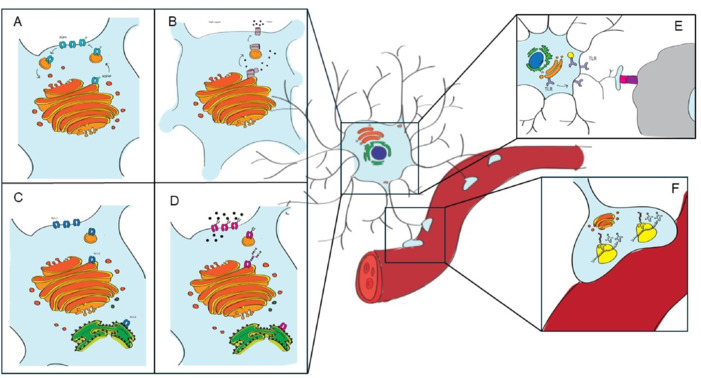
Examples of the role of the Golgi apparatus in astrocytes. The Golgi is in orange, and the Endoplasmic reticulum is in green. (A) Golgi and acquaporins 4 (in light blue). (B) Golgi and metal detoxification mediated by ATP7A (in light purple). (C) Golgi and Kv, different subtypes of Kv are in different compartments of the secretory pathway (Kv in blue). (D) Golgi and EAAT; glycosylated and non-glycosylated EAAT are in pink. (E) Golgi and neuroinflammation: TLR is in light purple. Astrocytes interact with target cells, and the TLR response happens inside the astrocyte. (F) Golgi and local translation; here, ribosomes are represented in yellow. ATP7A: Copper-transporting P-type ATPase; Kv: voltage–gated potassium channels; TLR: Toll-like receptor.

Typically, the GA is located close to the nucleus and the ER (Wei and Seemann, 2010). However, some interesting exceptions have been observed in highly polarized cells (Taverna et al., 2016). In some cases, as for the dendrites of neurons, the Golgi ribbon is absent from a cellular compartment, and GOs can be present to sustain local protein posttranslational modifications (Wang et al., 2020). GOs have been observed in astrocyte-long processes that end with endfeet. The endfeet contact the vasculature of the BBB and regulate BBB integrity, the crosstalk with the peripheral immune system, endothelial transport, and vessel contractility in response to neuronal activity and signaling. Boulay et al. (2017) demonstrated the presence of ribosome-bound mRNAs in the endfeet associated with the detection of nascent protein, suggesting a system for distal translation in astrocytes. The authors also showed that this feature was specific for mRNAs encoding for proteins of the endfeet, involved in processes connecting the BBB and inflammation, and influencing astrocyte-mediated homeostatic responses (Boulay et al., 2017; Sakers et al., 2017; **Figure 2F**). The combined presence of the translational machinery and the GO suggests that local protein translation/modification by GO may be crucial in astrocytes, influencing their interaction and homeostatic responses.

#### Golgi apparatus–mediated glycosylation regulates astrocytic functions

One of the main functions of GA is the post-translational modification of proteins and lipids. Glycosylation is one of the best-known modifications. It starts in the ER and continues throughout the Golgi stacks. Thus, GA creates a highly complex glycan signature, allowing proteins to be functional and providing a signal for their correct delivery. Alterations in the glycosylation machinery are associated with a plethora of diseases strongly affecting brain development and physiology. Impaired glycosylation ultimately causes misfunctioning of proteins, by altering protein folding and trafficking, as in the case of N-methyl-D-aspartate-type receptors whose expression is dramatically downregulated when N-glycosylation is inhibited (Scott and Panin, 2014).

In astrocytes, glycosylation can affect mitochondrial and inflammatory responses (Scott and Panin, 2014). Under pathological conditions, glia may secrete dysfunctional mitochondria with a signature of O-GalNacylation. GA also creates a highly complex glycan signature, and provides function, and a signal for their correct delivery. Inhibiting glycosylation indeed causes misfunctioning of proteins, such as in the case of N-methyl-D-aspartate-type receptors, whose expression is dramatically downregulated when N-glycosylation is inhibited (Scott and Panin, 2014).

Furthermore, in astrocytes, glycosylation can affect mitochondrial and inflammatory responses (Scott and Panin, 2014). Under pathological conditions, glia may secrete dysfunctional mitochondria with a signature of O-GalNacylation, exerting a neuroprotective action (Park et al., 2021a).

#### Golgi apparatus and glutamate transporter

Astrocytes are key in the uptake of glutamate released during synaptic transmission. EAAT2 is responsible for the majority of glutamate uptake in the cortex. Bauer et al. (2010) assessed the deglycosylation of EAAT1, EAAT2, and EAAT3 in schizophrenic patients. When their glycosylation is reduced, EAAT1 and EAAT2 are retained in the ER, decreasing their trafficking to the plasma membrane and glutamate transport (Conroy et al., 2021). This suggests that disorders in glycosylation of astrocytic glutamate transporters may be a feature of diseases, such as schizophrenia (Bauer et al., 2010; **Figure 2D**).

Another example of the relevance of glycosylation for astrocytic function is represented by dentin matrix protein 1, which is a critical factor for astrocyte maturation and BBB formation. Eradicating the glycosylation of dentin matrix protein 1 protein results in severe BBB disruption (Jing et al., 2018).

#### Golgi apparatus and voltage-gated potassium channels localization

Moreover, it has been observed that voltage-gated potassium channels (Kv) colocalize with the Golgi apparatus (Zhu et al., 2014). Kv is a class of proteins involved in the selective conduction of potassium ions, especially relevant for synaptic transmission. In astrocytes, two possible roles for Kv have been proposed: (1) Kv1.3 plays functions in the Golgi apparatus, regulating, for example, membrane potential, or (2) it constitutes a backup resource for plasma membrane Kv1.1 (Akhtar et al., 1999; Zhu et al., 2014; **Figure 2C**).

#### Golgi apparatus and metal detoxification

Astrocytes are key regulators of the homeostasis of the redox-active metals iron and copper in the brain. Astrocytes efficiently take up, store, and export copper, suggesting that these cells play a role in neuronal copper supply (Rathore et al., 2012; Li et al., 2024). Impaired Cu^2+^ supply from astrocytes to neurons has been observed in Menkes disease due to ATP7A mutations (De Bie et al., 2007). ATP7A is a copper-transporting P-type ATPase, and it undergoes copper-dependent trafficking between the trans-Golgi network and vesicular structures (**Figure 2B**). The Golgi is deeply involved in copper homeostasis by acting as a central hub for copper-dependent enzymes, such as GSH, and regulating copper levels within the cell (Scheiber and Dringen, 2011; Tiffany-Castiglioni et al., 2011; Dringen et al., 2013).

The GA plays an important role in metal detoxification by facilitating the transport, processing, and sorting of proteins involved in metal homeostasis and detoxification. Ion pumps, such as secretory pathway Ca^2+^-ATPase (SPCA), are located at the GA. Though primarily known for their role in calcium homeostasis, SPCA pumps also facilitate the transport and detoxification of manganese. These pumps are crucial for maintaining a sufficient supply of Mn^+^ for glycosyltransferases in the GA. Moreover, SPCA pumps prevent excessive accumulation of Ca^2+^ and Mn^2+^ in the cytosol, as this could trigger neurotoxicity and result in several neurological disorders (Rathore et al., 2012; Dringen et al., 2013; Boczek et al., 2021). Blocking SPCA activity with high levels of Mn^2+^ leads to GA fragmentation in glial cells and disrupts Ca^2+^ homeostasis. This is essential in cells such as astrocytes, where calcium signaling is vital in exerting homeostatic control. Furthermore, astrocytes are well known for their role in brain detoxification; thus, GA functionality becomes critical in ensuring astrocytes function properly.

Other toxic compounds, such as unconjugated bilirubin, may be a source of toxicity for astrocytes. Mrp1 is localized at the GA in cultured mouse astrocytes, and even low unconjugated bilirubin concentration rapidly upregulates its expression and translocation to the plasma membrane. This process protects astrocytes from cytotoxicity induced by unconjugated bilirubin (Dringen et al., 2013; Deng et al., 2018).

#### Golgi apparatus and ethanol detoxification

Ethanol-chronic exposure induces the retention of glycoproteins in growing astrocytes. Ethanol changes GA morphology and reduces the number of secretory vesicles. Blanco et al. (2008) have observed that in glia, ethanol leads to interleukin 1 receptor type I internalization in caveosomes enriched with lipid rafts and accumulation at the ER-Golgi and nucleus. This triggers downstream signaling pathways associated with inflammation (Blanco et al., 2008). In addition, ethanol-induced alteration of astrocyte secretome, suggesting an impact on the secretory pathway (Tomás et al., 2005; Ibáñez et al., 2021).

#### Golgi apparatus and aquaporins

Another relevant example of homeostatic function of astroglia is represented by aquaporin channels, which control and adjust water distribution across the membrane. Like other transmembrane proteins, AQP4 is transported to the GA to be phosphorylated and sorted into its destination. Golgi fragmentation may lead to impaired trafficking of AQP4 and a decrease in its degradation by disruption of AQP4 phosphorylation. Many CNS diseases have been observed concomitantly with mutations and overexpression of AQP4 in astrocytes, most notably resulting in BBB damage (Nicchia et al., 2004; Deng et al., 2018; Pham et al., 2024; **Figure 2A**).

#### The Golgi apparatus is involved in neuroinflammation

Astrocytes can secrete chemokines and cytokines, affecting the adaptive and innate immune responses. Toll-like receptor 4 (TLR4) is one member of the Toll-like receptors (TLRs) and, upon activation by pathogens, is translocated from the GA membrane, where it resides, to the plasma membrane. Dysfunction of the GA and the trafficking machinery impairs TRL4-mediated response and inflammation and influences the BBB integrity (**Figure 2E**). Ethanol-stimulated astrocytes undergo internalization of TLR4 receptors in caveosomes, thereby affecting the inflammatory response. Caveosomes are implicated in delivering cargo to the ER-Golgi apparatus and in the cellular nucleus (Latz et al., 2002; Blanco et al., 2008). Thus, the trafficking pathway and the Golgi play a key role in astrocyte-mediated neuroinflammation and response to brain damage. Moreover, astroglia can produce and secrete the C3a complement protein; C3a secretion is linked to GA integrity as it is disrupted upon brefeldin A treatment, a drug compound that induces GA fragmentation (Deng et al., 2018; Verkhratsky et al., 2023).

## Congenital Glycosylation Disorders: An Example of Golgi Apparatus Dysfunction and Future Perspective on the Possible Involvement of Astrocytes

**Figure 3:**
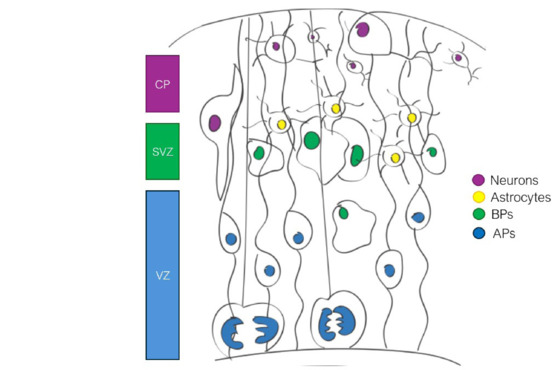
Neocortex development. Neocortex develops starting from APs (in blue) that divide at the apical surface and occupy the VZ. BPs (in green) occupy the SVZ. Both these cell types will give rise to the neurons (in purple), forming the six layers of the neocortex. Astrocytes (in yellow) develop from the cells in the SVZ. APs: Apical progenitors; BPs: basal progenitors; CP: cortical plate; SVZ: subventricular zone; VZ: ventricular zone.

GA is polarized in apical progenitors (APs), one of the first cell types that appear during brain development. They possess an apical process directed towards the ventricular surface and a basal process that contacts the basal lamina. APs receive different subsets of signals from two distinct environments that influence the cell identity and fate. During neocortex development, APs first undergo symmetric proliferative divisions to form the initial pool of cells to sustain the formation of the neocortex, and eventually give rise to another class of neural progenitor cells (NPCs), called basal progenitors (BPs) (Taverna et al., 2016; Arai and Taverna, 2017; Polenghi and Taverna, 2023). BPs will ultimately divide and form neurons and non-neuronal cells, such as astrocytes. Astrocytes are formed from NPCs in the region of the forming brain called the subventricular zone, which is positioned basally to the ventricular zone (VZ) (Clavreul et al., 2022). While APs reside in the VZ, BPs occupy the SVZ (**Figure 3**).

The sequence and timing of appearance of NPCs is a controlled process in which cells are formed at the right time in the right amount. Deregulation of this process is observed in neurodevelopmental diseases (Rasika et al., 2018).

Interestingly, in APs, the Golgi apparatus is restricted to the apical process (Taverna et al., 2016) but is absent from the basal process. This implies that two secretory pathways coexist in the same cell, to account for protein and lipid glycosylation: (i) a conventional route taking place in the apical process, and (ii) an unconventional route that allows proteins to be glycosylated and delivered to the plasma membrane even in the absence of GA. A reorganization of the GA is observed upon AP to BP fate switch (Taverna et al., 2016). When an AP gives rise to a BP, the Golgi apparatus undergoes a reorganization that brings GA to a perinuclear and pericentrosomal position.

Though the exact contribution of GA to the cell fate switch process is still not fully understood, a group of diseases named Congenital disorders of glycosylation (CDG) represents an important example of how mutation on one of the components of the Golgi apparatus can lead to neurodevelopmental disorders (Chang et al., 2018). In CDGs, mutations of Golgi resident proteins, Golgi matrix, or glycosylation enzymes, result in diseases with different severity, though the most affected organ is certainly the brain (Grunewald, 2002; Jaeken, 2010). Oftentimes in CDGs, primary microcephaly is observed as a clinical manifestation, suggesting a link between GA and brain development. For example, the conserved oligomeric Golgi complex, is involved in vesicle tethering between GA stacks and GA to ER, and mutations to subunits of this complex are associated with defects of the GA integrity and function which in turn very frequently translate into primary microcephaly, intellectual disability, and epilepsy, among other symptoms (Rasika et al., 2018; D’Souza et al., 2020).

Given the relevance of GA and glycosylation for astrocyte function, it is tempting to speculate that diseases of the CDG class could affect astrocyte function, but also, though indirectly, their formation. Given that astrocytes are formed by NPCs, CDGs or GA-related neurodevelopmental diseases may affect the formation of astrocytes, their number, functionality, and localization within the nascent neocortex. A recent work (Yale et al., 2023) shows that loss of MGAT5, an enzyme involved in glycosylation, causes an accelerated formation of neurons from NPCs, while the number is decreased in vitro. Furthermore, astrocytes have been studied for their role in many different CNS pathologies, including neurodegenerative diseases, neuropsychiatric disorders, intellectual disabilities, and epilepsy. Thus, it would be interesting to understand whether CDG-causing mutations of GA resident proteins could directly affect homeostatic function and defects of astrocytes, and cell-autonomus and non-cell autonomus related changes (Coulter and Steinhäuser, 2015; Wang et al., 2022; Verkhratsky et al., 2023).

## Conclusion

The Golgi apparatus and the secretory pathway may have a tight connection with homeostatic function of astrocytes. Indeed, astrocytes express and require for their proper plasticity a plethora of channels, receptors, and transporters. They need efficient machinery to balance ions, metals, water, and energy contents. Astrocytes contribute to neuronal function, providing neurotransmitters and their precursors, and clearing toxic metabolites. They provide support and protection from insults and pathogens, being the principal homeostatic cells of the CNS.

We reported that mutations to Golgi proteins are involved in controlling Golgi pH and linked to N- and O-glycosylation and trafficking defects. Polarized cells such as neurons and astrocytes represent an interesting example of differential positioning of the GA and GO within the cell to sustain local protein synthesis and posttranslational modifications. GA is involved in secretion, trafficking, and detoxification, and astrocytes extensively use the secretory pathway. Moreover, during CNS development, astrocytes are formed from NPCs in the region of the forming brain called the SVZ, which is positioned basally to the VZ. Alteration of GA may be involved in astrocyte differentiation. Given the relevance of GA and glycosylation for astrocyte function, a more comprehensive study on GA and secretory pathways in astrocytes is needed to clarify basic mechanisms of CNS physiology and diseases.

## Search Strategy

Studies mentioned in this review were searched on the https://pubmed.ncbi.nlm.nih.gov/ using the keywords: Golgi apparatus, trafficking, astrocytes, astroglial cells, secretory pathway, glycosylation and congenital glycosylation disorders.

## Data Availability

*Not applicable*.
